# Selective HDL-Raising Human Apo A-I Gene Therapy Counteracts Cardiac Hypertrophy, Reduces Myocardial Fibrosis, and Improves Cardiac Function in Mice with Chronic Pressure Overload

**DOI:** 10.3390/ijms18092012

**Published:** 2017-09-20

**Authors:** Ruhul Amin, Ilayaraja Muthuramu, Joseph Pierre Aboumsallem, Mudit Mishra, Frank Jacobs, Bart De Geest

**Affiliations:** Centre for Molecular and Vascular Biology, Department of Cardiovascular Sciences, Catholic University of Leuven, 3000 Leuven, Belgium; mdruhul.amin@kuleuven.be (R.A.); ilayaraja.muthuramu@kuleuven.be (I.M.); josephpierre.aboumsallem@kuleuven.be (J.P.A.); mudit.mishra@kuleuven.be (M.M.); FJACOBS1@its.jnj.com (F.J.)

**Keywords:** high-density lipoproteins, gene therapy, cardiac hypertrophy, heart failure, pressure overload, cardiac function, oxidative stress, apolipoprotein A-I

## Abstract

Epidemiological studies support an independent inverse association between high-density lipoprotein (HDL) cholesterol levels and heart failure incidence. The effect of selective HDL-raising adeno-associated viral serotype 8-human apolipoprotein (apo) A-I (AAV8-A-I) gene transfer on cardiac remodeling induced by transverse aortic constriction (TAC) was evaluated in C57BL/6 low-density lipoprotein receptor-deficient mice. Septal wall thickness and cardiomyocyte cross-sectional area were reduced by 16.5% (*p* < 0.001) and by 13.8% (*p* < 0.01), respectively, eight weeks after TAC in AAV8-A-I mice (*n* = 24) compared to control mice (*n* = 39). Myocardial capillary density was 1.11-fold (*p* < 0.05) higher and interstitial cardiac fibrosis was 45.3% (*p* < 0.001) lower in AAV8-A-I TAC mice than in control TAC mice. Lung weight and atrial weight were significantly increased in control TAC mice compared to control sham mice, but were not increased in AAV8-A-I TAC mice. The peak rate of isovolumetric contraction was 1.19-fold (*p* < 0.01) higher in AAV8-A-I TAC mice (*n* = 17) than in control TAC mice (*n* = 29). Diastolic function was also significantly enhanced in AAV8-A-I TAC mice compared to control TAC mice. Nitro-oxidative stress and apoptosis were significantly reduced in the myocardium of AAV8-A-I TAC mice compared to control TAC mice. In conclusion, selective HDL-raising human apo A-I gene transfer potently counteracts the development of pressure overload-induced cardiomyopathy.

## 1. Introduction

High-density lipoproteins (HDLs) consist of several subclasses, which share the presence of apolipoprotein (apo) A-I, phospholipids, and cholesterol but are distinct by the variable abundance of one or more representatives of at least 85 proteins and hundreds of lipid species [1,2,3]. HDLs are circulating multimolecular platforms that exert divergent functions: reverse cholesterol transport, anti-inflammatory effects, anti-oxidative effects, immunomodulatory effects, improved endothelial function, increased endothelial progenitor cell number and function, antithrombotic effects, and potentiation of insulin secretion and improvement of insulin sensitivity [4]. Pleiotropic effects of HDL might be translated into clinically significant effects in strategically selected therapeutic areas that are not directly related to native coronary artery disease [1,2,3].

Epidemiological studies support a strong association between HDL cholesterol levels and heart failure incidence. In Framingham Heart Study participants free of coronary heart disease at baseline, low HDL cholesterol levels were independently associated with heart failure incidence after adjustment for interim myocardial infarction and clinical covariates [5]. Apo A-I is the main apo of HDL, and apo A-I levels strongly correlate with cholesterol levels. Human apo A-I gene transfer inhibits the development of diabetic cardiomyopathy in rats [6]. HDL-raising gene transfer improves diastolic function in hypercholesterolemic mice, as indicated by an increased peak rate of isovolumetric relaxation [7]. Furthermore, human apo A-I gene transfer increases survival, reduces infarct expansion, attenuates left ventricular dilatation, and enhances systolic and diastolic cardiac function post-myocardial infarction in mice [8]. However, because beneficial effects of HDL-raising gene therapy on cardiac function post-ligation of the left anterior descending coronary artery may be dependent on improved infarct healing and reduced infarct expansion, direct effects of HDL on the ventricular wall cannot be proven in this model of myocardial infarction. 

Transverse aortic constriction (TAC) is a commonly used model for pressure overload-induced cardiac hypertrophy and heart failure [9]. TAC initially leads to compensatory hypertrophy of the heart, but over time, the response to chronic hemodynamic overload becomes maladaptive and results in cardiac dilatation and heart failure. The general hypothesis investigated in the current study is whether HDL-targeted human apo A-I gene therapy counteracts the development of non-ischemic cardiomyopathy and heart failure in mice. Specifically, we evaluated the effect of hepatocyte-specific adeno-associated viral (AAV) serotype 8-mediated human apo A-I gene therapy (AAV8-A-I) on the cardiac structure and function and on the development of heart failure in C57BL/6 low-density lipoprotein receptor (LDLr)-deficient mice subjected to pressure overload.

## 2. Results

### 2.1. Selective High-Density Lipoprotein (HDL)-Raising Gene Therapy Decreases Mortality after Transverse Aortic Constriction (TAC)

**Figure 1:**
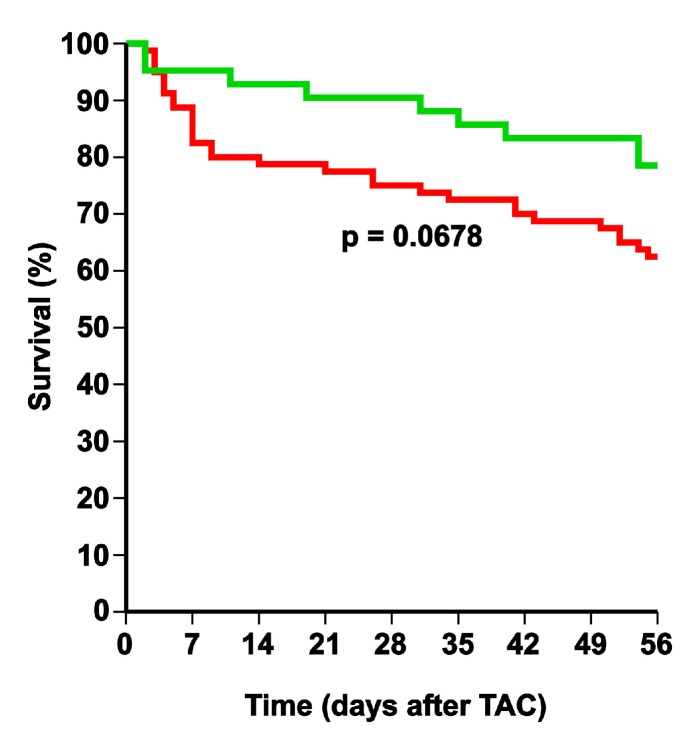
Comparison of Kaplan–Meier survival curves during an eight week follow-up period after transverse aortic constriction (TAC). Control TAC mice (red line) and adeno-associated viral serotype 8-human apolipoprotein A-I (AAV8-A-I) TAC mice (green line) are compared. The 0 day time-point corresponds to the induction of TAC at the age of 14 weeks. Survival analysis was performed by log-rank test.

HDL-raising gene transfer was performed at the age of 12 weeks in male C57BL/6 LDLr^−/−^ mice by the tail vein injection of 5 × 10^10^ genome copies of an AAV8 vector containing a hepatocyte-specific expression cassette to induce the expression of human apo A-I (AAV8-A-I). Two weeks later, TAC or sham operation was performed. AAV8-A-I gene transfer induced persistent and stable human apo A-I expression for the entire duration of the experiment. Human apo A-I levels at 10 weeks after gene transfer were 1730 ± 100 µg/mL (*n* = 10) in sham mice and 1780 ± 120 µg/mL (*n* = 10) in TAC mice. AAV8-A-I gene therapy increased HDL cholesterol levels 1.47-fold (*p* < 0.001) in sham mice and 1.45-fold (*p* < 0.001) in TAC mice, whereas non-HDL cholesterol levels were not significantly altered (Table 1). A comparison of Kaplan–Meier survival curves showed a clear trend (*p* = 0.0678) for a lower mortality rate in AAV8-A-I TAC mice compared to control TAC mice (hazard ratio for mortality of 0.543; 95% confidence interval (CI): 0.282 to 1.05) during eight weeks of follow-up (Figure 1). Sham operation did not result in any mortality (data not shown).

### 2.2. Atrial Hypertrophy, Lung Congestion, and Right Ventricular Hypertrophy in Control TAC Mice but Not in AAV8-A-I TAC Mice

No significant difference in body weight, organ weights, or tibia length was observed between the different sham groups (Table 2). Heart weight was increased 1.66-fold (*p* < 0.001) in control TAC mice and 1.52-fold (*p* < 0.001) in AAV8-A-I TAC mice, compared to respective sham groups. Equivalent differences were observed for the heart weight/tibia length ratio (Table 2). Similarly, left ventricular weight was increased 1.87-fold (*p* < 0.001) in control TAC mice and 1.65-fold (*p* < 0.001) in AAV8-A-I TAC mice, compared to respective sham groups. Right ventricular weight was 1.33-fold (*p* < 0.001) higher in control TAC mice than in control sham mice but was not significantly elevated in AAV8-A-I TAC mice compared to AAV8-A-I sham mice (Table 2). Atrial weight was 2.05-fold (*p* < 0.001) higher in control TAC mice than in control sham mice, whereas no significant alteration was observed in AAV8-A-I TAC mice compared to AAV8-A-I sham mice. Atrial weight was 45.7% (*p* < 0.05) lower in AAV8-A-I TAC mice compared to control TAC mice. Lung weight was 1.13-fold (*p* < 0.05) higher in control TAC mice than in control sham mice, whereas lung weight was not elevated at all in AAV8-A-I TAC mice. Lung weight was 13.3% (*p* < 0.05) lower in AAV8-A-I TAC mice compared to control TAC mice. Taken together, these data indicate left ventricular failure in control TAC mice, as evidenced by increased atrial weight, lung congestion, and right ventricular hypertrophy. In contrast, no evidence of heart failure was present in AAV8-A-I TAC mice.

### 2.3. AAV8-A-I Gene Transfer Counteracts Cardiac Hypertrophy, Increases Capillary Density and Relative Vascularity, and Reduces Interstitial and Perivascular Fibrosis after TAC

**Figure 2:**
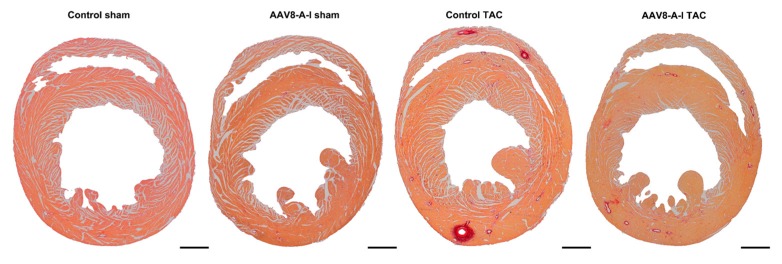
Representative Sirius red-stained cross-sections of sham hearts and transverse aortic constriction (TAC) hearts at day 56 after operation. Scale bar represents 1 mm.

**Figure 3:**
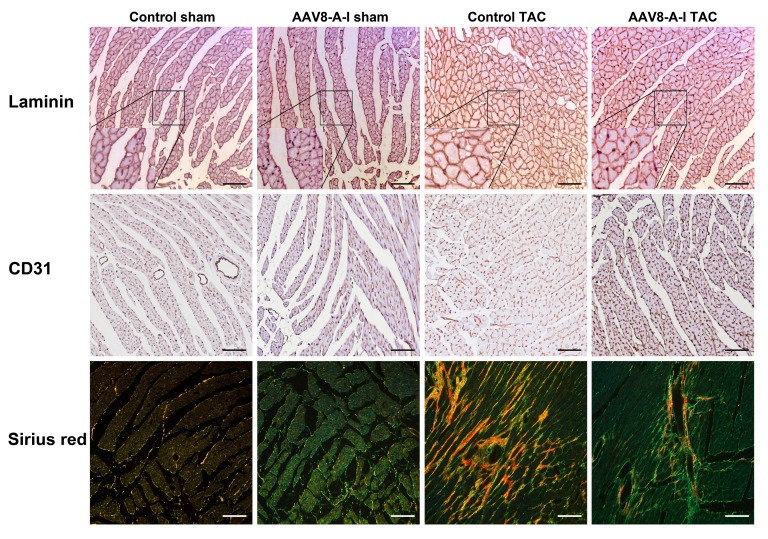
(Immuno)histochemical analysis of the myocardium of sham mice and transverse aortic constriction (TAC) mice at day 56 after operation. Representative photomicrographs show laminin-stained cardiomyocytes, CD31-positive capillaries, and Sirius red-stained interstitial collagen viewed under polarized light. Scale bar represents 50 μm. Insets show a 4× magnification of the boxed region.

Representative Sirius red-stained cross-sections of sham hearts and TAC hearts at day 56 after operation are shown in Figure 2. TAC induced a significant increase of the left ventricular wall area, anterior wall thickness, and septal wall thickness in both control TAC mice and in AAV8-A-I TAC mice, compared to respective sham groups (Table 3). However, left ventricular hypertrophy was less pronounced in AAV8-A-I TAC mice than in control TAC mice, as evidenced by a lower ventricular wall area (*p* < 0.05), septal wall thickness (*p* < 0.001), and anterior wall thickness (*p* < 0.05; Table 3). In addition, the cardiomyocyte cross-sectional area was 13.8% (*p* < 0.01) lower in AAV8-A-I TAC mice than in control TAC mice. The capillary density and relative vascularity were 1.11-fold (*p* < 0.05) and 1.10-fold (*p* < 0.05) higher, respectively, in AAV8-A-I TAC mice than in control TAC mice (Table 3). A prominent decrease of interstitial (*p* < 0.01) and perivascular fibrosis (*p* < 0.001) was observed in AAV8-A-I TAC mice compared to control TAC mice (Table 3). Figure 3 shows representative photomicrographs of laminin-stained cardiomyocytes, CD31-positive capillaries, and Sirius red-stained interstitial collagen viewed under polarized light. Taken together, AAV8-A-I gene therapy potently counteracts pathological structural remodeling after TAC. Coronary atherosclerosis was completely absent in all experimental groups (data not shown). 

### 2.4. Selective HDL-Raising Gene Therapy Improves Cardiac Function in Both Sham Mice and TAC Mice

AAV8-A-I gene therapy significantly improved diastolic function in sham mice, as evidenced by a 1.19-fold (*p* < 0.05) increase of the absolute value of the peak rate of isovolumetric contraction and a 14.5% (*p* < 0.05) reduction of the time constant of isovolumetric relaxation (Table 4). A prominent and better cardiac function was observed in AAV8-A-I TAC mice compared to control TAC mice. The end-diastolic pressure was significantly (*p* < 0.05) lower in AAV8-A-I TAC mice than in control TAC mice. Selective HDL-raising gene transfer significantly improved systolic cardiac function in TAC mice, as evidenced by a 1.18-fold (*p* < 0.01) increase of the peak rate of isovolumetric contraction. Diastolic function in AAV8-A-I TAC mice was significantly better than in control TAC mice, as evidenced by a 1.17-fold (*p* < 0.05) increase of the absolute value of the peak rate of isovolumetric relaxation and a 15.4% (*p* < 0.05) decrease of the time constant of isovolumetric relaxation. Taken together, cardiac function is significantly improved following AAV8-A-I gene transfer in both sham mice and TAC mice.

### 2.5. AAV8-A-I Gene Transfer Reduces Oxidative Stress and Myocardial Apoptosis after TAC

**Figure 4:**
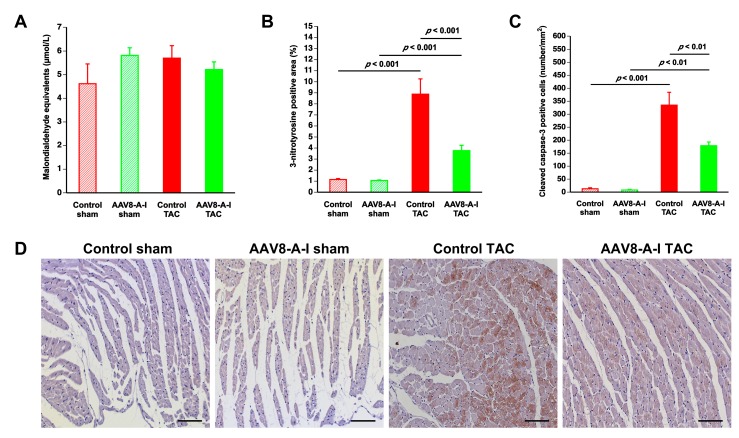
Quantification of oxidative stress and cardiomyocyte apoptosis in sham mice and in transverse aortic constriction (TAC) mice at day 56 after operation. Sham mice and TAC mice are indicated by open bars and closed bars, respectively. Plasma thiobarbituric acid-reactive substances (TBARS) expressed as plasma malondialdehyde equivalents, the percentage of 3-nitrotyrosine-positive myocardial area, and cleaved caspase-3-positive cells in the myocardium are shown in panels (**A**–**C**), respectively. Panel (**D**) illustrates representative photomicrographs showing myocardial sections stained for 3-nitrotyrosine. All data represent means ± SEM (*n* = 10). Scale bar represents 50 µm.

Plasma thiobarbituric acid-reactive substances (TBARS) and the 3-nitrotyrosine-positive area (%) in the myocardium are shown in Figure 4A, B, respectively. No differences in plasma TBARS were observed between different groups. Compared to respective sham groups, the 3-nitrotyrosine-positive area (%) in the myocardium quantified by immunohistochemistry was increased 7.73-fold (*p* < 0.001) and 3.57-fold (*p* < 0.001) in control TAC mice and in AAV8-A-I TAC mice, respectively. The 3-nitrotyrosine-positive area was 57.6% (*p* < 0.001) lower in AAV8-A-I TAC mice than in control TAC mice, indicating decreased nitro-oxidative stress. Apoptosis in the myocardium was evaluated using immunohistochemical quantification of cleaved caspase-3. Cleaved caspase-3-positive cells were scarce in the myocardium of sham mice (Figure 4C). Compared to control TAC mice, the number of cleaved caspase-3-positive cells was reduced by 46.7% (*p* < 0.01) in AAV8-A-I TAC mice (Figure 4C). Representative myocardial sections immunostained for 3-nitrotyrosine are shown in Figure 4D.

## 3. Discussion

The main findings of the present study are the following: (1) selective HDL-raising gene therapy exerts anti-hypertrophic effects under conditions of pressure overload; (2) AAV8-A-I gene transfer counteracts features of pathological hypertrophy following pressure overload in mice, as evidenced by reduced myocardial interstitial and perivascular fibrosis, an increased capillary myocardial density, and reduced apoptosis in the myocardium; (3) selective HDL-raising gene therapy improves diastolic cardiac function in both sham mice and in TAC mice and enhances systolic cardiac function in TAC mice; (4) pulmonary congestion and right ventricular hypertrophy are observed in control TAC mice, indicating the presence of left ventricular failure, which appears to be absent in AAV8-A-I TAC mice.

C57BL/6 LDLr^−/−^ mice were chosen as a model, as the lipoprotein distribution in LDLr-deficient mice, characterized by a predominance of apo B containing lipoproteins, resembles the human lipoprotein distribution significantly more closely than wild-type mice, characterized by a preponderance of HDL [10].

In the current study, a xenogeneic protein, human apo A-I, is expressed in mice. We have consistently observed the absence of a humoral and cellular immune response against human apo A-I in several murine strains after gene transfer with vectors containing a hepatocyte-specific expression cassette [11,12,13,14]. Human apo A-I gene transfer induces significant qualitative changes in HDL. We have previously shown that murine apo A-I levels decreased to less than 25% of baseline levels following human apo A-I gene transfer [15], which is consistent with data on human apo A-I transgenic mice [16]. This represents a post-transcriptional effect, as there is no decline of murine apo A-I mRNA levels [16]. Therefore, an important effect of human apo A-I gene transfer is the replacement of murine apo A-I with human apo A-I. Secondly, control mice expressing murine apo A-I only contain a monodisperse population of HDL particles with an average diameter of approximately 9.5 nm [16,17]. In contrast, transgenic mice expressing human apo A-I exhibit a polydisperse population of HDL particles [16]. Consistent with these observations in transgenic mice, HDL particles following human apo A-I gene transfer in mice were polydisperse with average diameters of 11, 9.7, 8.5 and 7.4 nm [18].

The vector applied in the current study contains a hepatocyte-specific expression cassette, which restricts transgene expression to parenchymal liver cells. Human α_1_-antitrypsin is only expressed in hepatocytes and macrophages, and the short version of the human α_1_-antitrypsin promoter does not contain sequences to induce expression in macrophages [19]. The α_1_-microglobulin/bikunin precursor enhancer is located 2.7 kb downstream from its promoter and is hepatocyte-specific [20,21,22]. The hepatic control region-1, previously described by Simonet et al. [23], is also hepatocyte-specific. All the effects in the current study are systemic effects reflecting secretion of human apo A-I by parenchymal liver cells.

Selective HDL-raising gene therapy exerted anti-hypertrophic effects on the myocardium under conditions of pressure overload. HDL has been shown to downregulate the angiotensin II type 1 receptor [24,25]. Furthermore, HDL inhibits mechanical stress-induced autophagy and hypertrophy in cultured cardiomyocytes [26]. Continuous infusion of HDL has been shown to inhibit cardiac hypertrophy in vivo [25,26], which may be mediated at least in part via downregulation of the angiotensin II type 1 receptor. 

Overexpression of human apo A-I in mice has been shown to increase the activity of two lipoprotein-associated enzymes, platelet-activating factor acetylhydrolase and paraoxonase, which are critical for the anti-inflammatory and anti-oxidative potential of HDL [27]. An increased anti-inflammatory and anti-oxidative potential of HDL may contribute to the beneficial effects of AAV8-A-I gene transfer on cardiac remodeling after TAC. Features of pathological hypertrophy are fibrotic remodeling, capillary rarefaction, and cardiomyocyte death, which promote cardiac dysfunction and development of heart failure [28]. Reactive oxygen species and oxidative stress contribute significantly to the pathophysiology of heart failure, having an impact on many key aspects of the failing heart phenotype, such as hypertrophy, matrix remodeling, contractile dysfunction, arrhythmia, and cell viability [29]. HDL-raising gene transfer in TAC mice reduced nitro-oxidative stress in the heart and decreased apoptosis in the myocardium. 

A prominent finding in the current study is that selective HDL-raising gene transfer enhances diastolic function in sham mice. The observed effect of AAV8-A-I gene transfer on the diastolic function in sham mice should be considered as a pharmacological effect that results in a supernormal diastolic function. This finding is consistent with our previous observations on the effect of gene transfer with an EAE3E4-deleted human apo A-I adenoviral vector on the cardiac function in female C57BL/6 LDLr^−/−^ mice [7]. The effect on the diastolic function occurred in the absence of detectable effects on the cardiac structure and it is compatible with direct electrophysiological effects of HDL. Reconstituted HDL shortens repolarization in isolated rabbit cardiomyocytes [30]. Moreover, the infusion of reconstituted HDL decreases the heart rate-corrected QT interval on surface electrocardiograms in humans [30]. A prolonged QT interval is useful to predict left ventricular diastolic dysfunction [31]. Taken together, the effects of HDL on the action potential and surface electrocardiogram are consistent with the effects of raised HDL on diastolic function. The systolic function and diastolic function were improved in AAV8-A-I TAC mice, compared to control TAC mice. Enhanced cardiac function in these mice was at least in part due to the attenuation of pathological cardiac remodeling.

A clear distinction should be made between cardiac dysfunction and heart failure. Heart failure in humans is defined as a clinical syndrome characterized by typical symptoms (e.g., breathlessness and fatigue) that may be accompanied by signs (e.g., elevated jugular venous pressure, pulmonary crackles, and peripheral oedema) caused by structural and/or functional cardiac abnormalities, resulting in a reduced cardiac output and/or elevated intracardiac pressures at rest or during stress [32]. This definition of heart failure restricts itself to stages at which clinical symptoms are apparent. Because subjective evidence of disease cannot be evaluated in animals, diagnosis of heart failure in mice is entirely dependent on objective clinical signs. The presence of increased wet lung weight indicating pulmonary congestion can be used for an operational diagnosis of heart failure in mice. Increased lung weight in control TAC mice but not in AAV8-A-I TAC mice is compatible with the absence of congestive heart failure in the latter. The presence of increased atrial weight in control TAC mice but not in AAV8-A-I TAC mice also suggests the presence of chronically elevated filling pressures in the former.

Left ventricular pressure overload after TAC initially induces reactive interstitial fibrosis and is subsequently followed by replacement fibrosis in areas of cardiomyocyte cell death [33]. Perivascular inflammation and fibrosis may decrease tissue availability to oxygen and nutrients and increase the pathological remodeling response [34]. Selective HDL-raising gene therapy potently inhibited interstitial and perivascular fibrosis after TAC. HDL has been shown to reduce transforming growth factor-ß1-induced collagen deposition in murine fibroblasts [35]. Furthermore, HDL has been demonstrated to decrease transforming growth factor-ß1-induced endothelial-mesenchymal transition in aortic endothelial cells in vitro [36]. An improved anti-oxidative and anti-inflammatory potential of HDL after AAV8-A-I gene therapy may explain the reduced perivascular fibrosis in AAV8-A-I TAC mice.

The current study was restricted to male mice, because significant sex differences in the degree of left ventricular hypertrophy after TAC exist [37,38]. This sex difference would increase variability within the same experimental group and would decrease the statistical power of a study in which mice of both sexes are compared. Moreover, sex differences in mice do not predict sex differences in humans. The observed effects in the current study are strong. It is reasonable to assume that the data on improved cardiac structure and function following AAV8-A-I gene transfer are robust and are therefore reproducible in female mice or in other genetic backgrounds. Clearly, interaction effects between treatment on the one hand and sex or genetic background on the other hand cannot be entirely excluded.

AAV8-mediated gene transfer has been used successfully to treat haemophilia B in a clinical trial [39]. AAV vectors have a theoretical potential for clinical translation. Therefore, this study represents an important step forward in comparison with previous HDL-raising gene therapy studies [40,41,42,43,44].

In conclusion, the current study demonstrates that HDL exerts beneficial effects on cardiac structure and function in the absence of coronary artery disease. Selective HDL-raising gene therapy improves cardiac function both in the absence and in the presence of pressure overload and counteracts adverse ventricular remodeling in mice with pressure overload. These data indicate that HDL-targeted therapies may have a potential for the prevention and treatment of non-ischemic cardiomyopathy.

## 4. Materials and Methods 

### 4.1. In Vivo Experiments Evaluating the Effect of HDL-Raising Gene Transfer on the Development of Pressure Overload-Induced Cardiomyopathy

All experimental procedures in animals were performed in accordance with protocols approved by the Institutional Animal Care and Research Advisory Committee of the Catholic University of Leuven (approval number: P154/2013, 1 October 2013). Male C57BL/6 LDLr^−/−^ mice, originally purchased from Jackson Laboratories (Bar Harbor, ME, USA), were fed a standard chow (SC) diet (Sniff Spezialdiäten GMBH, Soest, Germany) following weaning and for the entire duration of the experiment. Gene transfer was performed at the age of 12 weeks. HDL-raising gene transfer was performed by the tail vein injection of 5 × 10^10^ genome copies of an AAV8 vector containing a hepatocyte-specific expression cassette to induce the expression of human apo A-I (AAV8-A-I). The expression cassette of this vector consists of the 1272 bp *DC172* promoter, comprising an 890 bp α_1_-antitrypsin promoter fused together with two copies of the 160 bp *α_1_-microglobulin* enhancer [45], upstream of the 2 kb genomic human *apo* A-I sequence, one copy of the 774 bp *hepatic control region-1*, and the rabbit β-globin polyadenylation signal (127 bp). AAV vector production was performed as described [46]. The control AAV8-null vectors contained the transcriptional regulatory sequences but no genomic human *apo* A-I insert. 

To induce pressure overload, TAC was performed at the age of 14 weeks as described [47]. Briefly, anesthesia was performed with a single intraperitoneal injection of sodium pentobarbital (Nembutal, Ceva Sante Animale, Brussels, Belgium) at a dose of 40–70 mg/kg. The mice were put in a supine position and the temperature was maintained at 37 °C with a heating pad. A horizontal skin incision of 0.5 to 1 cm in length was made at the level of the suprasternal notch. A 2 to 3 mm longitudinal cut was made in the proximal portion of the sternum, and the thymus gland was retracted. This allowed for visualization of the aortic arch under low-power magnification. A wire with a snare at the end was passed under the aorta between the origin of the right innominate artery and the left common carotid artery. A 7-0 silk suture (Ethicon, Johnson & Johnson, Livingston, Scotland, UK) was snared with the wire and pulled back around the aorta. Subsequently, a bent 27-gauge needle (BD Microlance, BD, Franklin Lakes, NJ, USA) was placed next to the aortic arch and the suture was snugly tied around the needle and the aorta. Afterwards, the needle was quickly removed. The skin was closed and the mice were allowed to recover on a warming pad until they were fully awake. The sham procedure was identical except that no constriction on the aorta was applied. Buprenorphine (Temgesic; Reckitt Benckiser Healthcare Ltd., Hull, UK) was administered at a dose of 0.1 mg/kg body weight subcutaneously for peri-operative pain relief. Postoperative analgesia was applied immediately following the intervention. The euthanasia of mice at day 56 after sham or TAC operation was performed by cervical dislocation.

Group assignment at the start of the study was performed at random. At the end of the study, data of all surviving mice were included in the analysis. Investigators who performed the endpoint analyses were blinded to the group allocation. The unblinding of animal numbers corresponding to specific allocation groups was performed at the completion of measurements. The total number of mice included in the current study was 166. This number comprised 22 control sham mice, 22 AAV8-A-I sham mice, 80 control TAC mice, and 42 AAV8-A-I TAC mice. No sham mice died during the experiment, whereas 30 control TAC mice and 9 AAV8-A-I TAC mice died.

### 4.2. In Vivo Hemodynamic Measurements

Invasive hemodynamic measurements were performed eight weeks after TAC or after sham operation as described [47]. The mice were anesthetized by the intraperitoneal administration of 1.4 g/kg urethane (Sigma, Steinheim, Germany). Body temperature was maintained with a heating pad and monitored with a rectal probe. An incision in the right carotid artery was made with a 26-gauge needle between a distal and proximal non-occlusive ligation of the artery. A 1.0 French Millar pressure catheter (SPR-67/NR; Millar instruments, Houston, TX, USA) was inserted and advanced to the left ventricle (LV). After the stabilization of the catheter, the heart rate, maximal systolic LV pressure, minimal diastolic LV pressure, peak rate of isovolumetric LV contraction (dP/dt_max_), and peak rate of isovolumetric LV relaxation (dP/dt_min_) were measured. The end-diastolic LV pressure was calculated manually from the pressure in the function of time curves. The time constant of isovolumetric LV pressure fall (tau) was calculated using the method of Weiss et al. [48]. Arterial blood pressure measurements were obtained after the withdrawal of the catheter from the LV to the ascending aorta. Data were registered with Powerlab Bridge Amplifier and Chart software (sampling rate of 2000 Hz; ADInstruments Ltd., Oxford, UK).

### 4.3. Blood Sampling

Blood (volume of 600–800 µL) was obtained by a puncture of the vena cava inferior at the end of the experiment just before euthanasia. Anticoagulation was performed with a 0.1 volume of 136 mmol/L trisodium citrate, and plasma was immediately isolated by centrifugation at 1100× *g* for 10 min and stored at −20 °C.

### 4.4. Plasma Lipoprotein Analysis

Plasma levels of total cholesterol and free cholesterol were determined using a Cholesterol Quantification kit from Sigma (Sigma, St. Louis, MO, USA). Lipoproteins were separated from 25 µL of citrate-anticoagulated plasma using a photopolymerized loading gel stained with Sudan black dye (Lipoprint, Los Angeles, CA, USA) [49]. The samples were subjected to electrophoresis for 60 min at 3 mA per gel tube, set at a maximum delivery of 500 V. Bands were identified on the basis of the migration distance, according to the instructions of the manufacturer.

### 4.5. Human Apo A-I Enzyme-Linked Immunosorbent Assay

Human apo A-I plasma levels were determined by an enzyme-linked immunosorbent assay (ELISA), according to the instructions of the manufacturer (Abcam, Cambridge Science Park, UK). Standards and samples were added to the wells, followed by the antibody mix. After incubation, the wells were washed to remove unbound material, and the chromogenic 3,3′,5,5′-tetramethylbenzidine substrate solution was added. In the presence of horseradish peroxidase enzyme conjugates, 3,3′,5,5′-tetramethylbenzidine and peroxide react, leading to the production of 3,3′,5,5′-tetramethylbenzidine diimine, which causes the solution to take a blue color with a maximum absorbance at 605 nm. The color intensity is proportional to the amount of horseradish peroxidase activity, which in turn is related to the levels of bound analyte and thus to the human apo A-I in the plasma. The addition of acidic stop solution changes the color from blue to yellow (absorbance maximum at 450 nm), which stabilizes the color development to enable accurate measurement of the intensity at 450 nm using a spectrophotometer. A blank was subtracted from the standards and samples. A standard curve was created by plotting the logarithm of the absorbance of each standard versus the logarithm of the soluble factor concentration. The correlation coefficient corresponding to the regression curve was 0.99 or higher. No cross-reaction with murine apo A-I was observed.

### 4.6. Analysis of Lipid Peroxidation in Plasma

Measurement of TBARS used for the quantification of lipid peroxidation was performed according to the instructions of the manufacturer (Cayman Chemical, Ann Arbor, MI, USA).

### 4.7. Histological and Morphometric Analysis

Histological and morphometric analyses were executed as described [47]. After hemodynamic analysis, the mice were perfused via the abdominal aorta with phosphate-buffered saline (PBS) and the hearts were arrested in diastole by CdCl_2_ (100 μL; 0.1 mol/L), followed by perfusion fixation with 1% paraformaldehyde in PBS. After dissection, the hearts were post-fixated overnight in 1% paraformaldehyde, embedded in paraffin, and 6 μm thick cross-sections at 130 μm spaced intervals were made extending from the apex to the basal part of the LV. LV remodeling was assessed by morphometric analysis on mosaic images of Sirius red-stained heart cross-sections using Axiovision 4.6 software (Zeiss, Zaventem, Belgium). Anterior wall thickness and septal wall thickness were determined. All geometric measurements were computed in a blinded fashion from representative tissue sections of four separate regions, and the average value was used to represent that animal for statistical purposes [8,50].

To measure collagen content in the interstitium, Sirius red staining was performed as described by Junqueira et al. [51]. Sirius red polarization microscopy on a Leica RBE microscope with KS300 software (Zeiss) was used to quantify thick, tightly packed mature collagen fibers as orange-red birefringent and loosely packed, less cross-linked and immature collagen fibers as yellow-green birefringent. Collagen-positive area was normalized to the LV remote area and was expressed as a percentage. Any perivascular fibrosis was excluded from this analysis. Perivascular fibrosis was quantified as the ratio of the fibrosis area surrounding the vessel to the total vessel area. Two mid-ventricular sections were studied per animal [47].

Cardiomyocyte hypertrophy was analyzed on paraffin sections stained with rabbit anti-mouse laminin (Sigma; 1/50) by measuring the cardiomyocyte cross-sectional area (μm^2^) of at least 200 randomly selected cardiomyocytes in the LV myocardium. The capillary density in the myocardium was determined on CD31-stained sections using rat anti-mouse CD31 antibodies (BD; 1/500). Relative vascularity in the myocardium was determined as (capillary density (number/mm^2^)/cardiomyocyte density (number/mm^2^))/(cardiomyocyte cross-sectional area (μm^2^)) [6]. Two mid-ventricular cross-sections were analyzed per mouse [8,50].

Immunostaining for 3-nitrotyrosine was performed with rabbit anti-nitrotyrosine antibodies (Merck Millipore, Overijse, Belgium; dilution of 1/250).

Apoptosis was quantified on deparaffinized tissue sections using a SignalStain-cleaved caspase-3 IHC detection kit (Cell Signaling Technologies, Beverly, MA, USA), which utilizes a polyclonal rabbit antibody to the neoepitope peptide at the end of cleaved caspase-3 [9].

### 4.8. Statistical Analysis

All data are expressed as means ± standard error of the means (SEM). Parameters between four groups were compared by one-way analysis of variance followed by Bonferroni multiple comparisons post-test for comparing sham groups, TAC groups, and sham versus respective TAC groups using GraphPad Instat (GraphPad Software, San Diego, CA, USA). When the assumption of sampling from populations with identical standard deviations was not met, a logarithmic transformation was performed. When the assumption of sampling from populations with Gaussian distributions was not met, a Kruskal–Wallis test was performed, followed by Dunn’s multiple comparison post-test. Parameters between two groups were compared using the Student’s *t*-test. When indicated, a logarithmic transformation or a non-parametric Mann–Whitney test was performed. The assumption of Gaussian distribution was tested using the Kolmogorov–Smirnov method. Kaplan–Meier survival curves were analyzed by a log-rank test using Prism4 (GraphPad Software, San Diego, CA, USA). A two-sided *p*-value of less than 0.05 was considered statistically significant.

## Figures and Tables

**Table 1:** Total, non-HDL, and HDL cholesterol plasma levels (mmol/L) in C57BL/6 LDLr^−/−^ mice 10 weeks after gene transfer.

Parameter	Control Sham	AAV8-A-I Sham	Control TAC	AAV8-A-I TAC
Number of mice	10	10	10	10
Total cholesterol	5.56 ± 0.33	5.96 ± 0.21	5.84 ± 0.34	6.01 ± 0.23
Non-HDL cholesterol	4.42 ± 0.33	4.28 ± 0.25	4.70 ± 0.30	4.36 ± 0.25
HDL cholesterol	1.14 ± 0.05	1.68 ± 0.08 °°°	1.14 ± 0.07	1.66 ± 0.09 ***

Data are expressed as means ± SEM (*n* = 10). °°° *p* < 0.001 versus control sham. *** *p* < 0.001 versus control TAC. HDL: high-density lipoproteins; TAC: transverse aortic constriction; AAV8-A-I: adeno-associated viral (AAV) serotype 8 vector containing a hepatocyte-specific human apo A-I expression cassette.

**Table 2:** Body weight and organ weights in C57BL/6 LDLr^−/−^ mice.

Parameter	Control Sham	AAV8-A-I Sham	Control TAC	AAV8-A-I TAC
Number of mice	10	10	11	9
Body weight (g)	28.8 ± 0.6	29.7 ± 0.5	27.5 ± 0.3	29.2 ± 0.9
Heart weight (mg)	135 ± 3	133 ± 7	224 ±18 ^§§§^	201 ± 7 ^§§§^
Heart weight/tibia length (mg/mm)	7.78 ± 0.12	7.66 ± 0.40	12.7 ± 1.0 ^§§§^	11.5 ± 0.4 ^§§§^
Left ventricular weight (mg)	86.9 ± 2.6	86.9 ± 5.0	163 ± 15 ^§§§^	143 ± 8 ^§§^
Right ventricular weight (mg)	22.7 ± 0.8	24.2 ± 0.9	30.1 ± 2.0 ^§§§^	27.9 ± 2.7
Atrial weight (mg)	9.10 ± 0.85	8.10 ± 0.92	18.6 ± 2.3 ^§§§^	10.1 ± 0.7 *
Lung weight (mg)	152 ± 3	151 ± 2	171 ± 8 ^§^	149 ± 3 *
Tibia length (mm)	17.4 ± 0.1	17.3 ± 0.1	17.5 ± 0.1	17.6 ± 0.1

Gene transfer was carried out in male C57BL/6 LDLr^−/−^ mice at the age of 12 weeks. TAC or sham operation was performed at the age of 14 weeks. Mice were sacrificed eight weeks later. ^§^ *p* <0 .05; ^§§^ *p* < 0.01; ^§§§^ *p* < 0.001 versus respective sham groups. * *p* < 0.05 versus control TAC. LDLr^−/−^: low-density lipoprotein receptor.

**Table 3:** Morphometric and histological parameters of the left ventricular myocardium in C57BL/6 LDLr^−/−^ mice.

Parameter	Control Sham	AAV8-A-I Sham	Control TAC	AAV8-A-I TAC
Number of mice	12	12	39	24
Left ventricular wall area (mm^2^)	10.4 ± 0.3	9.93 ± 0.30	14.2 ± 0.3 ^§§§^	13.1 ± 0.4 ^§§§,^*
Septal wall thickness (µm)	1110 ± 20	990 ± 20	1430 ± 30 ^§§§^	1190 ± 30 ^§§,^***
Anterior wall thickness (µm)	1130 ± 30	1090 ± 20	1420 ± 30 ^§§§^	1270 ± 30 ^§§,^*
Cardiomyocyte cross-sectional area (µm^2^)	228 ± 8	228 ± 12	502 ± 13 ^§§§^	433 ± 21 ^§§§,^**
Cardiomyocyte density (number/mm^2^)	4530 ± 180	4400 ± 160	2240 ± 60 ^§§§^	2680 ± 120 ^§§,^*
Capillary density (number/mm^2^)	6400 ± 170	6020 ± 290	5330 ± 150 ^§§^	5910 ± 220 *
Relative vascularity (µm^−2^)	0.00632 ± 0.00020	0.00610 ± 0.00026	0.00487 ± 0.00014 ^§§§^	0.00536 ± 0.00020 ^§,^*
Interstitial fibrosis (%)	0.939 ± 0.073	0.892 ± 0.083	9.98 ± 0.70 ^§§§^	5.46 ± 0.41 ^§§,^**
Perivascular fibrosis (ratio)	0.291 ± 0.010	0.287 ± 0.019	0.880 ± 0.022 ^§§§^	0.562 ± 0.031 ^§,^***

Gene transfer was carried out in male C57BL/6 LDLr^−/−^ mice at the age of 12 weeks. TAC or sham operation was performed at the age of 14 weeks. Mice were sacrificed eight weeks later. ^§^ *p* < 0.05; ^§§^ *p* < 0.01; ^§§§^ *p* < 0.001 versus respective sham groups. * *p* < 0.05; ** *p* < 0.01; *** *p* < 0.001 versus control TAC.

**Table 4:** Hemodynamic parameters in C57BL/6 LDLr^−/−^ mice.

Parameter	Control Sham	AAV8-A-I Sham	Control TAC	AAV8-A-I TAC
Number of mice	10	12	29	16
LEFT VENTRICLE				
Peak systolic pressure (mm Hg)	100 ± 2	102 ± 3	175 ± 5 ^§§§^	177 ± 6 ^§§§^
End-diastolic pressure (mm Hg)	2.91 ± 0.32	2.63 ± 0.34	4.04 ± 0.48	2.12 ± 0.39 *
dP/dt_max_ (mm Hg/ms)	11.9 ± 1.1	12.5 ± 0.7	10.2 ± 0.3	12.1 ± 0.4 **
dP/dt_min_ (mmHg/ms)	−9.32 ± 0.70	−11.1 ± 0.3 °	−10.3 ± 0.5	−12.1 ± 0.4 *
Tau (ms)	6.39 ± 0.43	5.47 ± 0.12 °	6.32 ± 0.27	5.35 ± 0.14 *
Heart rate (bpm)	559 ± 19	601 ± 14	597 ± 9	629 ± 8
AORTA				
Systolic pressure (mm Hg)	99.6 ± 2.5	101 ± 5	174 ± 6 ^§§§^	177 ± 7 ^§§§^
Diastolic pressure (mm Hg)	61.9 ± 1.9	63.5 ± 2.0	58.2 ± 2.9	64.4 ± 3.6
Mean pressure (mm Hg)	79.8 ± 2.1	81.1 ± 2.3	98.8 ± 2.9 ^§§^	105 ± 3 ^§§§^

Gene transfer was carried out in male C57BL/6 LDLr^−/−^ mice at the age of 12 weeks. TAC or sham operation was performed at the age of 14 weeks. Mice were sacrificed eight weeks later. ° *p* < 0.05 versus control sham; ^§§^ *p* < 0.01; ^§§§^ *p* < 0.001 versus respective sham groups. * *p* < 0.05; ** *p* < 0.01 versus control TAC.

## Abbreviations

AAV	Adeno-associated viral
HDL	High-density lipoproteins
apo A-I	Apolipoprotein A-I
TAC	Transverse aortic constriction
LV	Left ventricle
TBARS	Thiobarbituric acid-reactive substances

## References

[1] Shah A.S., Tan L., Lu Long J., Davidson W.S. (2013). The proteomic diversity of high density lipoproteins: Our emerging understanding of its importance in lipid transport and beyond. J. Lipid Res..

[2] Gordts S.C., Singh N., Muthuramu I., de Geest B. (2014). Pleiotropic effects of HDL: Towards new therapeutic areas for HDL-targeted interventions. Curr. Mol. Med..

[3] Muthuramu I., Amin R., de Geest B. (2017). New perspectives on biological HDL-targeted therapies. Expert Opin Biol. Ther..

[4] Van Linthout S., Frias M., Singh N., de Geest B. (2015). Therapeutic potential of HDL in cardioprotection and tissue repair. Handb. Exp. Pharmacol..

[5] Velagaleti R.S., Massaro J., Vasan R.S., Robins S.J., Kannel W.B., Levy D. (2009). Relations of lipid concentrations to heart failure incidence: The Framingham heart study. Circulation.

[6] Van Linthout S., Spillmann F., Riad A., Trimpert C., Lievens J., Meloni M., Escher F., Filenberg E., Demir O., Li J. (2008). Human apolipoprotein A-I gene transfer reduces the development of experimental diabetic cardiomyopathy. Circulation.

[7] Gordts S.C., van Craeyveld E., Muthuramu I., Singh N., Jacobs F., de Geest B. (2012). Lipid lowering and HDL raising gene transfer increase endothelial progenitor cells, enhance myocardial vascularity, and improve diastolic function. PLoS ONE.

[8] Gordts S.C., Muthuramu I., Nefyodova E., Jacobs F., van Craeyveld E., de Geest B. (2013). Beneficial effects of selective HDL-raising gene transfer on survival, cardiac remodelling and cardiac function after myocardial infarction in mice. Gene Ther..

[9] Muthuramu I., Singh N., Amin R., Nefyodova E., Debasse M., Van Horenbeeck I., Jacobs F., de Geest B. (2015). Selective homocysteine-lowering gene transfer attenuates pressure overload-induced cardiomyopathy via reduced oxidative stress. J. Mol. Med..

[10] Van Craeyveld E., Gordts S., Jacobs F., de Geest B. (2010). Gene therapy to improve high-density lipoprotein metabolism and function. Curr. Pharm. Des..

[11] De Geest B., van Linthout S., Collen D. (2001). Sustained expression of human apo A-I following adenoviral gene transfer in mice. Gene Ther..

[12] De Geest B.R., van Linthout S.A., Collen D. (2003). Humoral immune response in mice against a circulating antigen induced by adenoviral transfer is strictly dependent on expression in antigen-presenting cells. Blood.

[13] Feng Y., Jacobs F., Van Craeyveld E., Lievens J., Snoeys J., Van Linthout S., de Geest B. (2010). The impact of antigen expression in antigen-presenting cells on humoral immune responses against the transgene product. Gene Ther..

[14] Gordts S.C., van Craeyveld E., Jacobs F., de Geest B. (2011). Gene transfer for inherited metabolic disorders of the liver: Immunological challenges. Curr. Pharm. Des..

[15] Feng Y., van Eck M., Van Craeyveld E., Jacobs F., Carlier V., Van Linthout S., Erdel M., Tjwa M., de Geest B. (2009). Critical role of scavenger receptor-BI-expressing bone marrow-derived endothelial progenitor cells in the attenuation of allograft vasculopathy after human apo A-I transfer. Blood.

[16] Rubin E.M., Ishida B.Y., Clift S.M., Krauss R.M. (1991). Expression of human apolipoprotein A-I in transgenic mice results in reduced plasma levels of murine apolipoprotein A-I and the appearance of two new high density lipoprotein size subclasses. Proc. Natl. Acad. Sci. USA.

[17] Golder-Novoselsky E., Forte T.M., Nichols A.V., Rubin E.M. (1992). Apolipoprotein ai expression and high density lipoprotein distribution in transgenic mice during development. J. Biol. Chem..

[18] Feng Y., Van Craeyveld E., Jacobs F., Lievens J., Snoeys J., de Geest B. (2009). Wild-type apo A-I and apo A-I(Milano) gene transfer reduce native and transplant arteriosclerosis to a similar extent. J. Mol. Med..

[19] Perlino E., Cortese R., Ciliberto G. (1987). The human α 1-antitrypsin gene is transcribed from two different promoters in macrophages and hepatocytes. EMBO J..

[20] Rouet P., Raguenez G., Ruminy P., Salier J.P. (1998). An array of binding sites for hepatocyte nuclear factor 4 of high and low affinities modulates the liver-specific enhancer for the human α1-microglobulin/bikunin precursor. Biochem. J..

[21] Rouet P., Raguenez G., Tronche F., Mfou′ou V., Salier J.P. (1995). Hierarchy and positive/negative interplays of the hepatocyte nuclear factors HNF-1, -3 and -4 in the liver-specific enhancer for the human α-1-microglobulin/bikunin precursor. Nucleic Acids Res..

[22] Rouet P., Raguenez G., Tronche F., Yaniv M., N′Guyen C., Salier J.P. (1992). A potent enhancer made of clustered liver-specific elements in the transcription control sequences of human α 1-microglobulin/bikunin gene. J. Biol. Chem..

[23] Simonet W.S., Bucay N., Lauer S.J., Taylor J.M. (1993). A far-downstream hepatocyte-specific control region directs expression of the linked human apolipoprotein E and C-I genes in transgenic mice. J. Biol. Chem..

[24] Van Linthout S., Spillmann F., Lorenz M., Meloni M., Jacobs F., Egorova M., Stangl V., de Geest B., Schultheiss H.P., Tschope C. (2009). Vascular-protective effects of high-density lipoprotein include the downregulation of the angiotensin II type 1 receptor. Hypertension.

[25] Lin L., Gong H., Ge J., Jiang G., Zhou N., Li L., Ye Y., Zhang G., Ge J., Zou Y. (2011). High density lipoprotein downregulates angiotensin II type 1 receptor and inhibits angiotensin II-induced cardiac hypertrophy. Biochem. Biophys. Res. Commun..

[26] Lin L., Liu X., Xu J., Weng L., Ren J., Ge J., Zou Y. (2015). High-density lipoprotein inhibits mechanical stress-induced cardiomyocyte autophagy and cardiac hypertrophy through angiotensin II type 1 receptor-mediated PI3K/Akt pathway. J. Cell. Mol. Med..

[27] De Geest B., Stengel D., Landeloos M., Lox M., Le Gat L., Collen D., Holvoet P., Ninio E. (2000). Effect of overexpression of human apo A-I in C57BL/6 and C57BL/6 apo E-deficient mice on 2 lipoprotein-associated enzymes, platelet-activating factor acetylhydrolase and paraoxonase. Comparison of adenovirus-mediated human apo A-I gene transfer and human apo A-I transgenesis. Arterioscler. Thromb. Vasc. Biol..

[28] Shimizu I., Minamino T. (2016). Physiological and pathological cardiac hypertrophy. J. Mol. Cell. Cardiol..

[29] Hafstad A.D., Nabeebaccus A.A., Shah A.M. (2013). Novel aspects of ros signalling in heart failure. Basic Res. Cardiol..

[30] Den Ruijter H.M., Franssen R., Verkerk A.O., van Wijk D.F., Vaessen S.F., Holleboom A.G., Levels J.H., Opthof T., Sungnoon R., Stroes E.S. (2011). Reconstituted high-density lipoprotein shortens cardiac repolarization. J. Am. Coll. Cardiol..

[31] Wilcox J.E., Rosenberg J., Vallakati A., Gheorghiade M., Shah S.J. (2011). Usefulness of electrocardiographic qt interval to predict left ventricular diastolic dysfunction. Am. J. Cardiol..

[32] Ponikowski P., Voors A.A., Anker S.D., Bueno H., Cleland J.G., Coats A.J., Falk V., Gonzalez-Juanatey J.R., Harjola V.P., Jankowska E.A. (2016). 2016 ESC guidelines for the diagnosis and treatment of acute and chronic heart failure: The task force for the diagnosis and treatment of acute and chronic heart failure of the european society of cardiology (ESC). Developed with the special contribution of the heart failure association (HFA) of the ESC. Eur. J. Heart Fail..

[33] Travers J.G., Kamal F.A., Robbins J., Yutzey K.E., Blaxall B.C. (2016). Cardiac fibrosis: The fibroblast awakens. Circ. Res..

[34] Kai H., Mori T., Tokuda K., Takayama N., Tahara N., Takemiya K., Kudo H., Sugi Y., Fukui D., Yasukawa H. (2006). Pressure overload-induced transient oxidative stress mediates perivascular inflammation and cardiac fibrosis through angiotensin II. Hypertens. Res..

[35] Spillmann F., de Geest B., Muthuramu I., Amin R., Miteva K., Pieske B., Tschope C., Van Linthout S. (2016). Apolipoprotein A-I gene transfer exerts immunomodulatory effects and reduces vascular inflammation and fibrosis in ob/ob mice. J. Inflamm..

[36] Spillmann F., Miteva K., Pieske B., Tschope C., van Linthout S. (2015). High-density lipoproteins reduce endothelial-to-mesenchymal transition. Arterioscler. Thromb. Vasc. Biol..

[37] Witt H., Schubert C., Jaekel J., Fliegner D., Penkalla A., Tiemann K., Stypmann J., Roepcke S., Brokat S., Mahmoodzadeh S. (2008). Sex-specific pathways in early cardiac response to pressure overload in mice. J. Mol. Med..

[38] Kararigas G., Fliegner D., Forler S., Klein O., Schubert C., Gustafsson J.A., Klose J., Regitz-Zagrosek V. (2014). Comparative proteomic analysis reveals sex and estrogen receptor β effects in the pressure overloaded heart. J. Proteome Res..

[39] Nathwani A.C., Tuddenham E.G., Rangarajan S., Rosales C., McIntosh J., Linch D.C., Chowdary P., Riddell A., Pie A.J., Harrington C. (2011). Adenovirus-associated virus vector-mediated gene transfer in hemophilia B. N. Engl. J. Med..

[40] Van Craeyveld E., Lievens J., Jacobs F., Feng Y., Snoeys J., de Geest B. (2009). Apolipoprotein A-I and lecithin: Cholesterol acyltransferase transfer induce cholesterol unloading in complex atherosclerotic lesions. Gene Ther..

[41] Van Craeyveld E., Gordts S.C., Nefyodova E., Jacobs F., de Geest B. (2011). Regression and stabilization of advanced murine atherosclerotic lesions: A comparison of LDL lowering and HDL raising gene transfer strategies. J. Mol. Med..

[42] Li R., Chao H., Ko K.W., Cormier S., Dieker C., Nour E.A., Wang S., Chan L., Oka K. (2011). Gene therapy targeting LDL cholesterol but not HDL cholesterol induces regression of advanced atherosclerosis in a mouse model of familial hypercholesterolemia. J. Genet. Syndr. Gene Ther..

[43] De Geest B., Zhao Z., Collen D., Holvoet P. (1997). Effects of adenovirus-mediated human apo A-I gene transfer on neointima formation after endothelial denudation in apo E-deficient mice. Circulation.

[44] Tangirala R.K., Tsukamoto K., Chun S.H., Usher D., Pure E., Rader D.J. (1999). Regression of atherosclerosis induced by liver-directed gene transfer of apolipoprotein A-I in mice. Circulation.

[45] Jacobs F., Snoeys J., Feng Y., Van Craeyveld E., Lievens J., Armentano D., Cheng S.H., De Geest B. (2008). Direct comparison of hepatocyte-specific expression cassettes following adenoviral and nonviral hydrodynamic gene transfer. Gene Ther..

[46] Lock M., Alvira M., Vandenberghe L.H., Samanta A., Toelen J., Debyser Z., Wilson J.M. (2010). Rapid, simple, and versatile manufacturing of recombinant adeno-associated viral vectors at scale. Hum. Gene Ther..

[47] Muthuramu I., Amin R., Postnov A., Mishra M., Jacobs F., Gheysens O., van Veldhoven P.P., de Geest B. (2017). Coconut oil aggravates pressure overload-induced cardiomyopathy without inducing obesity, systemic insulin resistance, or cardiac steatosis. Int. J. Mol. Sci..

[48] Weiss J.L., Frederiksen J.W., Weisfeldt M.L. (1976). Hemodynamic determinants of the time-course of fall in canine left ventricular pressure. J. Clin. Investig..

[49] McLaren D.G., Wang S.P., Stout S.J., Xie D., Miller P.L., Mendoza V., Rosa R., Castro-Perez J., Previs S.F., Johns D.G. (2013). Tracking fatty acid kinetics in distinct lipoprotein fractions in vivo: A novel high-throughput approach for studying dyslipidemia in rodent models. J. Lipid Res..

[50] Van Craeyveld E., Jacobs F., Gordts S.C., de Geest B. (2012). Low-density lipoprotein receptor gene transfer in hypercholesterolemic mice improves cardiac function after myocardial infarction. Gene Ther..

[51] Junqueira L.C., Bignolas G., Brentani R.R. (1979). Picrosirius staining plus polarization microscopy, a specific method for collagen detection in tissue sections. Histochem. J..

